# A Cross-Sectional Study Exploring the Relationship between Work-Related, Lifestyle Factors and Non-Specific Neck and Shoulder Pain in a Southeast Asian Population

**DOI:** 10.3390/healthcare12181861

**Published:** 2024-09-15

**Authors:** Chi Ngai Lo, Victoria Yu En Teo, Nur Farah Ain Binte Abdul Manaff, Tessa Chu-Yu Seow, Karthik Subramhanya Harve, Bernard Pui Lam Leung

**Affiliations:** 1 Family Care Physiotherapy Clinic, 154 West Coast Road, West Coast Plaza, 01-86, Singapore 127371, Singapore; 2 Health and Social Sciences Cluster, Singapore Institute of Technology, 10 Dover Drive, Singapore 138683, Singaporekarthik.harve@singaporetech.edu.sg (K.S.H.)

**Keywords:** neck and shoulder pain, computer, smartphone usage, post-COVID-19

## Abstract

Background and Objectives: Non-specific neck and shoulder pain (NSNSP) is prevalent among working adults. The increased use of electronic devices and prevalence of remote working and study following the COVID-19 pandemic have raised concerns about the potential rise in such conditions. This study aims to investigate the associations between work-related, lifestyle factors and NSNSP in the adult Southeast Asian Singaporean population. Materials and Methods: An online survey was administered electronically to Singaporeans aged 21 and above. Demographic data, NSNSP prevalence, computer and smartphone usage durations, sleep patterns, and exercise frequency were captured after obtaining informed consent (SIT institutional review board approval #2023014). Results: A total of 302 validated responses were recorded, including 212 suffering from NSNSP versus 90 in the comparison group. The NSNSP group showed significantly longer smartphone usage (5.37 ± 3.50 h/day) compared to the comparison group (4.46 ± 3.36 h/day, *p* = 0.04). Furthermore, the NSNSP group had lower exercise frequency (2.10 ± 1.74 days/week vs. 2.93 ± 2.21 days/week, *p* < 0.01) and shorter weekly exercise duration (2.69 ± 3.05 h/week vs. 4.11 ± 4.15 h/week, *p* < 0.01). The average NSNSP severity in this group was 34.9 ± 19.96 out of 100, correlating significantly with age (r = 0.201, *p* < 0.01) and BMI (r = 0.27, *p* < 0.01). Conclusions: This preliminary cross-sectional study examines characteristics of adult Southeast Asians with NSNSP post-COVID-19 pandemic. The findings indicate significantly longer smartphone use and less exercise in NSNSP respondents, with both age and body mass index (BMI) demonstrating significant correlations with NSNSP severity.

## 1. Introduction

Neck and shoulder pain are highly prominent musculoskeletal complaints, with a prevalence of over 20% among university students in Hong Kong [1,2]. In Singapore, a cross-sectional study reported a prevalence of neck pain of 23% among individuals aged 37–67 [3]. Neck and shoulder pain have been significantly associated with computer/smartphone usage, prolonged sitting, and static posture [4,5,6]. In addition, neck and shoulder pain are also linked to depressive symptoms and represent a significant burden on medical costs [7,8]. These factors highlight the importance of addressing work-related and lifestyle factors in understanding and managing non-specific neck and shoulder pain (NSNSP).

Neck and shoulder pain can be non-specific, presenting without definite structural abnormalities. Recent systematic reviews suggest that the structural integrity of shoulders in an ultrasound scan or magnetic resonance imaging may not necessarily be associated with pain and disability [9,10,11]. A working diagnosis of non-specific neck and shoulder pain (NSNSP) is mostly based on self-report assessment and physical tests [12,13,14]. Management of NSNSP also focuses more on the symptoms and impairment rather than the underlying pathophysiology, with the actual mechanism of the condition and treatment responsiveness remaining unclear.

Neck and shoulder pain are associated with prolonged computer usage with odds ratios (ORs) of 1.3–2.5 [4]. Prolonged static or improper postures (OR 1.74–2.44) [6,15] and improper position of computer equipment (OR 1.76–2.18) [16] have been identified as significant risk factors for neck and shoulder pain. Besides the mechanical factors mentioned above, it is intriguing that other additional factors including insufficient rest (<7 h; OR = 3.2), depressive signs (OR = 6.14), and smoking (OR = 8.99) are strongly correlated with NSNSP in young adults [8,15,17]. Also, uninterrupted prolonged sitting may significantly reduce cerebral blood flow and increase blood pressure in desk workers, as suggested by recent studies [18,19]. This points to the question that NSNSP is associated with both general metabolism and computer use and/or static posture inducing mechanical stress. The last cross-sectional study in Singapore was conducted before the pandemic period of coronavirus-19 (COVID-19) [3]. During the pandemic, the adoption of digital technologies in Singapore accelerated significantly, necessitating a policy framework for universal digital access [20]. Measures included the implementation of distance learning across various levels of education [21,22] and work-from-home arrangements for several working groups [23]. According to Rangaswamy et al. (2024), data showed that more than 70% of employees were working from home in Singapore during the study period [23].

Therefore, we aim to investigate the potential associations between the work-related, lifestyle factors post-COVID-19 and the presence of NSNSP in an adult Southeast Asian Singaporean population through a cross-sectional study.

## 2. Materials and Methods

A cross-sectional study was conducted from 30 January 2023 to 31 October 2023 and administered via online questionnaire to an adult population in Singapore through convenience sampling methods. It was managed using the Qualtrics survey system (Qualtrics, Provo, UT, USA), ensuring secure and efficient data collection. Participants were informed about the survey through multiple channels, including internal emails from the Singapore Institute of Technology, public advertisements on Facebook, and direct invitations via the researchers’ personal contacts including emails, WhatsApp messages, and other personal social media. Respondents accessed the Qualtrics survey questions through instructions provided in these announcements, enabling them to complete the survey anonymously while ensuring confidentiality and data integrity. 

The target population was adults aged 21 and above. Responses from individuals with and without neck and shoulder pain were compared. Individuals with prior acute or chronic injuries and pre-existing medical conditions relating to the cervical spine and shoulders were excluded. The first page of the online questionnaire provided information regarding the objectives and background of this study, privacy, and the informed consent statement. Participation in this study was voluntary, and the details of the questionnaire can be found in Appendix A. Ethical approval was granted by the Institutional Review Board (IRB) of the Singapore Institute of Technology (IRB approval number 2023014) prior to data collection. 

### Questionnaire Design

The questionnaire was meticulously designed by the authors to capture a comprehensive set of data related to demographic information, health and medical history, lifestyle factors, physical activity, pain symptoms, and cardiovascular health. This structured approach ensured that all relevant variables associated with NSNSP were considered. Established validity and reliability for this specific questionnaire are not available since non-standardized assessment tools were used. However, most of the questions were adapted from previous studies [1,3,24], providing a reasonable basis for the validity and relevance of the questions. The specific domains and questions included in the questionnaire are elaborated below.

Demographic Data: Participants were asked to provide their age in years, gender, height in centimeters, and weight in kilograms (Q1–4). This basic demographic information is crucial for understanding the general characteristics of the sample population and for adjusting analyses to account for potential confounding variables. Specific screening questions (Q5–8) were designed to screen out potential neck and shoulder pain due to known injuries or diseases rather than overuse from computer or smartphone usage or prolonged sitting.

Work-Related Factors: The questionnaire included detailed questions about participants’ daily work habits that could influence the prevalence of NSNSP. Participants reported the total duration of computer use in a day, the total duration of smartphone use in a day, and the average hours spent working or studying in a sitting position daily (Q9–11). These were the main quantifiable outcomes of this study to find out any correlations with NSNSP.

Pain and Symptoms: Participants were questioned about the presence of neck or shoulder pain, with a simple Yes/No question (Q17). Those who reported pain were then asked to rate the severity of their pain on a scale from 0 to 100, with 0 indicating no pain and 100 indicating the worst pain imaginable (Q18). This self-reported pain measure is an important outcome to test for correlation with other factors.

Physical Activities: To gauge participants’ physical activity levels, the questionnaire asked about the number of days they exercised per week and the duration of exercise per day (Q15–16). Participants were also asked if they participated in regular sports activities or exercises and how many hours per week they engaged in aerobic exercises (Q19–21). According to previous systematic reviews, physical activities including strengthening, aerobic, or general physical activities are effective in managing neck and shoulder pain in office workers, with standardized mean differences of 0.37 and an overall effect size of 3.8, respectively [25,26].

General Health: Participants were asked questions related to their sleeping duration (Q12), smoking habits, specifically the number of cigarettes smoked daily (Q13), and their alcohol consumption (Q14). Insufficient rest and smoking are strongly correlated with NSNSP in young adults [15,17]. Additionally, participants were asked about their history of cardiovascular diseases, any family history of such conditions, and any current medications they were taking (Q22–24). This information helps identify any underlying cardiovascular issues that might correlate with NSNSP.

Detailed questions can be found in Appendix A.

## 3. Sample Size Calculation and Statistical Analysis

The sample size was calculated by GPower 3.1.9.3 (Universitat Kiel, Kiel, Germany). To detect the correlation between the health-related characteristics and the severity of NSNSP, assuming an alpha level of 0.05, a statistical power of 0.8 for a two-tailed test, and a moderate correlation (p H1 = 0.3), a total of 138 subjects were required. Data were analyzed using SPSS (version 23, IBM Corp Ltd., Armonk, NY, USA). Independent *t*-tests and Chi-Squared tests were conducted to compare the characteristics of the responses from individuals with or without NSNSP. The Independent *t*-test was used to compare the mean values between two independent groups. It was employed to determine whether there is a statistically significant difference in the means of a continuous variable (e.g., smartphone usage duration, exercise frequency) between two unrelated groups. Similarly, the Chi-Squared test was used to assess if there was any significant difference in the categorical variables such as gender or drinking habits between the two groups. 

Pearson Correlation analysis was computed to assess any association between the severity of NSNSP and the variables of the duration of computer use and sitting, duration of exercise, and sleeping time. This test was used to evaluate the strength and direction of the linear relationship between two continuous variables (e.g., severity of neck pain and age, BMI). It is indicated when the goal is to measure the degree to which two continuous variables are related. To further explore the influence of potential covariates including the role of age and gender, regression and multiple regression analyses were conducted. Analysis of Covariance (ANCOVA) and partial correlation analyses were employed to adjust confounding factors to isolate the effects of the primary variables on the outcomes. 

In SPSS, Levene’s Test for Equality of Variances was used to check the assumption of equal variances, and adjusted significance values were also provided.

## 4. Results

A total of 632 responses were initially received. However, 330 responses were excluded for the following reasons: meeting exclusion criteria (e.g., recent or chronic injuries to the neck or shoulder region, pre-existing medical conditions related to the cervical spine and shoulders), missing major responses (such as the duration of computer use, smartphone use, sitting duration, and presence of neck or shoulder pain), irrelevant values (e.g., typing nonsensical letters or symbols instead of numbers), missing most demographic data, or extreme values. In total, 302 valid responses were analyzed, comprising 212 individuals suffering from NSNSP and 90 in the comparison group.

Although the initial sample size calculation determined that 138 subjects were required, we received a total of 302 responses. This larger sample size not only meets but exceeds our initial requirements, thereby increasing the reliability and validity of our results. Among the responders, 212 individuals suffering from NSNSP and 90 responses reported the absence of NSNSP (thereby serving as the comparison group). The details and statistical analysis of the responses are presented in Table 1. The average age of the valid respondents was approximately 31.45 years. Both groups had more female respondents than males, but the NSNSP group had a significantly higher female ratio (*p* = 0.02). The two groups demonstrated similarities in several demographic and lifestyle factors, including age, body mass index (BMI), computer use, sitting duration, sleep duration, daily exercise, drinking habits, and history of cardiovascular disease (Table 1).

Importantly, we observed smartphone usage duration, with the NSNSP group (5.37 ± 3.50 h/day) showing significantly longer usage compared to the comparison group (4.46 ± 3.36 h/day) by nearly an hour daily (*p* = 0.04). Weekly exercise duration was calculated by multiplying exercise frequency (days/week) by daily exercise duration (hours/day). Notably, the NSNSP group exhibited significantly lower exercise frequency (2.10 ± 1.74 days/week vs. 2.93 ± 2.21 days/week, *p* < 0.01) and shorter weekly exercise duration (2.69 ± 3.05 h/week vs. 4.11 ± 4.15 h/week, *p* < 0.01) compared to the subjects without neck pain. The average computer use of the respondents was about 6 h per day, the average sitting duration was between 6 and 7 h per day, and the average sleep duration was around 6.5 h per day. Due to the limited number of smokers (only 11 in total), statistical analysis pertaining to smoking behaviors was not feasible in this study.

Additional regression analysis indicated that gender (R^2^ = 0.03, *p* < 0.01), exercise frequency (R^2^ = 0.04, *p* < 0.01), and weekly exercise duration (R^2^ = 0.04, *p* < 0.05) have significant associations with the presence of NSNSP, while smartphone use (R^2^ = 0.01, *p* = 0.07) and other factors do not have significant effects.

ANCOVA was conducted to examine the effect of age, gender, and the presence of NSNSP on other factors (Table 2). Age had a significant effect on smartphone usage (F = 5.08, *p* = 0.03), exercise frequency (F = 13.30, *p* < 0.01), daily and weekly exercise duration (F = 6.19 to 17.64, *p* ≤ 0.01), and family history of cardiovascular disease (F = 6.43, *p* = 0.01). On the other hand, gender had a significant effect on exercise frequency (F = 13.30, *p* < 0.01), daily and weekly exercise duration (F = 6.97 to 7.64, *p* = 0.01–0.02), drinking habits (F = 4.50, *p* = 0.04), and history of cardiovascular disease (F = 5.66, *p* = 0.02). While regression showed associations between weekly exercise duration and NSNSP, the ANCOVA results suggest that smartphone usage and weekly exercise duration may not be significantly different between the NSNSP and the comparison group after controlling for age and gender. Exercise frequency remained a significant factor of the presence of NSNSP (F = 8.55, *p* < 0.01) after adjusting for age and gender.

**Figure 1:**
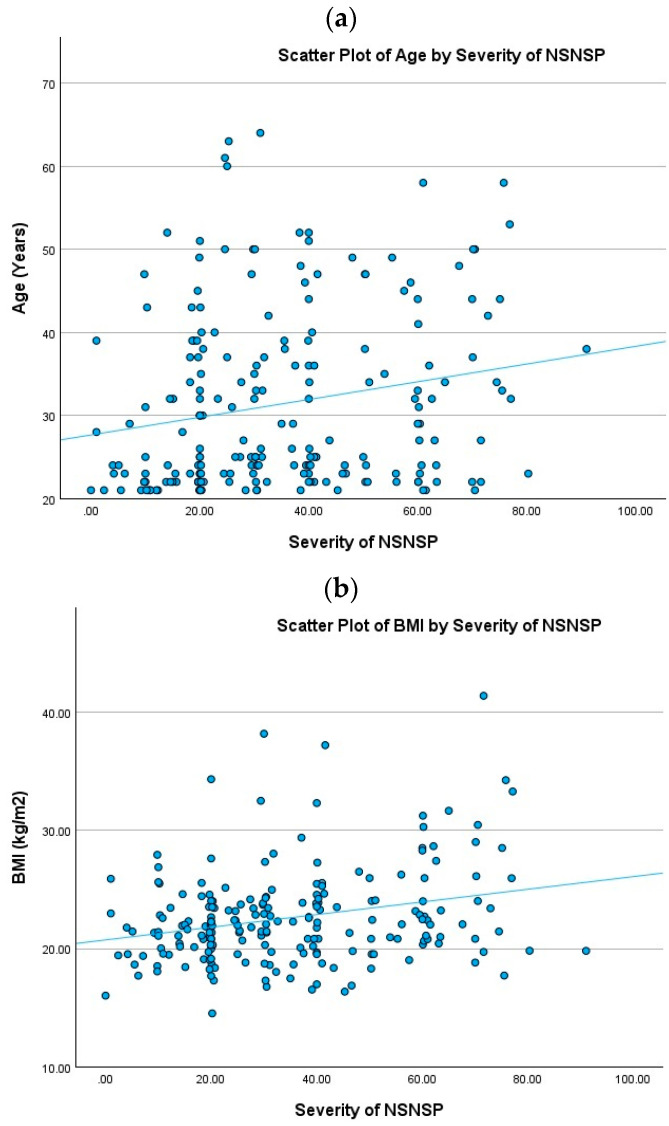
Lack of correlation between age and BMI with severity of NSNSP. (**a**) scatter plot of age against the severity of NSNSP (r = 0.201, *p* < 0.01), (**b**) scatter plot of BMI against the severity of NSNSP (r = 0.266, *p* < 0.01).

The average severity of neck and shoulder pain in the NSNSP group was 34.9 ± 19.96 out of 100. Among all the items, the severity of the pain was found to correlate significantly with age (r = 0.201, *p* < 0.01) and BMI (r = 0.266, *p* < 0.01) only (Table 3, Figure 1a,b). The statistics reflected that the severity of the pain had no significant correlations with the duration of smartphone usage, computer usage, sitting, or exercise. 

The regression analysis indicated that age, drinking, history of cardiovascular disease, and family history of cardiovascular disease do not have significant effects on pain severity. Furthermore, the multiple regression analysis did not reveal any additional significant results beyond those identified in the regression analysis. Partial correlation analysis, after adjusting for age and gender, also showed no additional significant results in the outcomes either. 

## 5. Discussion

Our study provides updated information regarding NSNSP among Southeast Asian adults from Singapore after the COVID-19 pandemic. The results suggest that gender, exercise frequency, and weekly exercise duration may have certain associations with the presence of NSNSP. The comparison group without NSNSP reported significantly more exercise time than the NSNSP population, with the NSNSP group averaging 2.69 h per week compared to 4.11 h per week in the comparison group. In addition, age and BMI were found to have statistically significant correlations with NSNSP severity. While the NSNSP group exhibited significantly longer smartphone usage (5.37 h per day) compared to the comparison group (4.46 h per day), the statistical correlation between smartphone usage and the presence or severity of NSNSP remains unclear.

The ANCOVA analysis revealed that age and gender played a significant role in influencing smartphone usage, exercise habits, and cardiovascular history. Younger individuals tended to use smartphones more frequently, while gender differences were most pronounced in exercise habits and cardiovascular disease history. These findings suggest that while demographic factors such as age and gender are related to differences in lifestyle behaviors, only exercise frequency remained a significant factor associated with the presence of NSNSP after adjusting for age and gender.

Our study’s findings align with previous studies which also identified gender as a significant risk factor for NSNSP [3,27]. Possible explanations for this gender difference may be due to lower neck muscle volume in women and biological factors like hormonal variations affecting pain sensitivity [3]. These factors may contribute to the higher number of females with NSNSP in our study. However, it is important to note that our study is not a case–control trial. Thus, the higher female response rate to our survey does not necessarily imply a higher prevalence of NSNSP in females in our population. In our comparison group, there were also more female respondents than male. Therefore, while our findings suggest a trend on gender, these should be interpreted with caution due to potential allocation errors inherent in convenience sampling.

In previous studies, Moreira-Silva et al. (2016) and Chen et al. (2018) have summarized seven and five articles, respectively, to show that physical activity and strengthening exercises are effective in managing NSNSP in office workers, with standardized mean differences of 0.37 and overall effect of 3.8, respectively [25,26]. Among all these studies, exercises including strengthening, aerobic, or general physical activities are effective for shoulder and neck pain in office workers. Those studies propose that regular physical activity helps improve muscle strength, flexibility, and postural control and improve blood circulation, reduce inflammation, and enhance the overall resilience of neck and shoulder muscles [25,26]. Interestingly, although the NSNSP group had a significantly lower exercise duration and frequency in this study, a negative correlation between exercise and the severity of NSNSP was not present. 

Our study is a cross-sectional study; therefore, it is not possible to establish causal relationships between exercise and NSNSP [28,29]. To draw causal inferences, a longitudinal study with an appropriate follow-up period would be necessary [30]. For instance, a randomized controlled trial conducted by Sihawong et al. demonstrated that a 12-month exercise program effectively reduced the incidence of neck pain (hazard rate = 0.45, 95% CI 0.28 to 0.71), though it did not significantly correlate with pain intensity [31]. In contrast, a longitudinal study in Norway involving nearly 30,000 vocationally active subjects found that exercise was associated with a lower relative risk of neck pain, though the results were not statistically significant [32]. Our findings align with these studies, suggesting that more exercise time may result in lower risk of NSNSP.

Out data suggest BMI has a statistically significant but weak correlation with the severity of NSNSP (0.2 < r < 0.3) [33]. Obesity and overweightness are well-known proinflammatory metabolic disorders relating to high levels of inflammatory markers including interleukin (IL-)1β, IL-6, C-reactive protein, and tumor necrosis factor (TNF)α [34,35]. Specifically, a meta-analysis indicated increased IL-1β (SMD: 0.84 [95% CI 0.24, 1.44], *p* = 0.01, I2 = 59%) and TNFα (SMD: 0.59 [0.09, 1.09], *p* = 0.02, I2 = 45%) in patients with chronic neck pain [36], which may explain the correlation between BMI and the severity of NSNSP shown in this study.

Neck pain could commonly be due to overuse or inflammatory changes involving neck muscles such as the sub-occipital muscles, as well as semispinalis and splenius that attach to the transverse and spinous processes of the vertebrae as well as inflammation affecting the intervertebral ligaments and the intervertebral joints [37,38]. In addition, inflammatory changes may also be associated with chronic conditions such as ankylosing spondylosis, rheumatoid arthritis leading to degeneration of the vertebrae, and arthritic lesions as well. Neck pain may also result from lesions involving the various parts of the cervical spine in both chronic (inflammations) and acute settings (trauma) [38,39]. Posterior herniation of the intervertebral disc (disc prolapse) may impinge on the spinal nerves exiting the vertebral canal, resulting in chronic pain, often a result of prolonged staring at the computer as an occupational hazard leading to disc degeneration as well as fatigue or spasm of the muscles [38,40]. Stretching and degeneration of muscles attaching the cervical spine to the shoulder girdle such as the trapezius and rhomboids may lead to chronic shoulder pain that may well be associated with neck pain and stiffness [41].

Compared with the previously published study by Hey et al. (2021), our study included a larger sample size in the NSNSP group. While Hey et al. (2021) reported a median age of 52 years in their study population [3], the respondents in our study were from a younger cohort with an average age of approximately 31 years. Furthermore, our study captured detailed data regarding average sleep duration, computer use, smartphone use, sitting time, and exercise duration among Singaporeans post-COVID-19 pandemic, which were not reported in the previous study. These data may be useful as benchmarks for future Singapore healthcare studies.

### Limitations

There are several limitations in this study. The overall response rate and validated responses collected in the study can be improved. The normality of the distribution for continuous variables was assessed using the Shapiro–Wilk test. Although the Shapiro–Wilk tests indicate that some data have a deviation from normality, the large sample size (n > 138) justifies the use of parametric tests such as the Independent *t*-test and Pearson Correlation, as supported by the Central Limit Theorem [42].

Previous literature suggests that depressive signs have a significant correlation with NSNSP in young people [8]. However, we did not include questions regarding the mental health status of respondents, which represents a limitation of this study. Finally, considering the increase in frequency of cyber scamming activities in Singapore post-COVID-19 pandemic [43], this study did not capture the details of sociodemographic and occupational characteristics to avoid low response rates, which could have compromised the overall sample size and the quality of the data.

## 6. Conclusions

This study examined a cohort of adult Southeast Asian Singaporeans with NSNSP post-COVID-19 pandemic. The results indicated that respondents with NSNSP reported significantly longer durations of smartphone use and less exercise frequency and time compared to those without NSNSP. Additionally, our data suggest that both age and BMI are significantly correlated with the severity of NSNSP.

## Figures and Tables

**Table 1:** Characteristics between the NSNSP group and comparison group.

	NSNSP Group	Comparison Group	Independent *t*-Test/Chi-Square Test
Age	*n* = 212 31.49 ± 10.57	*n* = 90 31.34 ± 13.67	*p* = 0.93
Gender (Male/Female) *^#^	Male: 45 (21.2%) Female: 163 (76.9%) Non-binary/Prefer not to say: 4 (1.9%)	Male: 34 (37.8%) Female: 55 (61.1%) Non-binary/Prefer not to say: 1 (1.1%)	* *p* = 0.02
BMI (kg/m^2^)	*n* = 212 22.66 ± 4.05	*n* = 90 22.55 ± 3.83	*p* = 0.82
Computer use (h/day)	*n* = 203 6.0 ± 3.54	*n* = 88 5.8 ± 3.65	*p* = 0.67
Smartphone use (h/day) *	*n* = 205 5.37 ± 3.50	*n* = 89 4.46 ± 3.36	* *p* = 0.04
Sitting position (h/day)	*n* = 204 6.96 ± 3.37	*n* = 89 6.34 ± 3.44	*p* = 0.15
Sleep (h/day)	*n* = 208 6.49 ± 1.33	*n* = 88 6.47 ± 1.66	*p* = 0.88
Exercise frequency (days/week) *	*n* = 211 2.10 ± 1.74	*n* = 89 2.93 ± 2.21	* *p* < 0.01
Daily exercise duration (h/day)	*n* = 205 0.97 ± 0.78	*n* = 87 1.08 ± 0.81	*p* = 0.442
Weekly exercise duration (h/week) *	*n* = 205 2.69 ± 3.05	*n* = 87 4.11 ± 4.15	* *p* < 0.01
Aerobic exercise (h/week)	*n* = 127 2.16 ± 1.96	*n* = 64 2.64 ± 2.93	*p* = 0.24
Drinking	*n* = 212 Non-drinker = 87 (41%) Social drinker = 114 (53.8%) Drink weekly = 9 (4.2%) Drink daily = 2 (0.9%)	*n* = 90 Non-drinker = 44 (48.9%) Social drinker = 42 (46.7%) Drink weekly = 3 (3.3%) Drink daily = 1 (1.1%)	*p* = 0.65
History of cardiovascular disease	Yes = 23 (10.8%) No = 173 (88.2%)	Yes = 14 (15.6%) No = 75 (84.4%)	*p* = 0.35
Family history of cardiovascular disease	Yes = 76 (35.8%) No = 120 (64.2%)	Yes = 29 (32.2%) No = 60 (67.8%)	*p* = 0.32

Legend: values are presented as mean ± standard deviation; * statistically significant, *p* < 0.05; ^#^ 5 cases indicated as non-binary or prefer not to disclose.

**Table 2:** Results of ANCOVA of the key variables after adjusting for age and gender associated with NSNSP.

Dependent Variable	Factor	Partial Eta Squared	F-Value	*p*-Value
Computer use (h/day)	Age	0.00	0.04	0.85
	Gender	0.00	0.35	0.56
	Presence of NSNSP	0.00	0.27	0.60
Smartphone use (h/day)	* Age	0.02	5.08	0.03 *
	Gender	0.01	2.20	0.14
	Presence of NSNSP	0.01	3.40	0.07
Sitting position (h/day)	Age	0.01	2.65	0.10
	Gender	0.00	0.06	0.80
	Presence of NSNSP	0.01	1.98	0.16
Sleep (h/day)	Age	0.00	0.24	0.62
	Gender	0.00	0.94	0.33
	Presence of NSNSP	0.00	0.09	0.77
Exercise frequency (days/week)	* Age	0.02	6.11	0.01 *
	* Gender	0.04	13.3	<0.01 *
	* Presence of NSNSP	0.03	8.55	<0.01 *
Daily exercise duration (h/day)	* Age	0.02	6.19	0.01 *
	* Gender	0.03	7.46	0.01 *
	Presence of NSNSP	0.00	0.48	0.49
Weekly exercise duration (h/week)	* Age	0.06	17.64	<0.01 *
	* Gender	0.02	6.97	0.02 *
	Presence of NSNSP	0.00	6.97	0.57
Aerobic exercise (h/week)	Age	0.00	0.59	0.45
	Gender	0.01	1.28	0.26
	Presence of NSNSP	0.01	1.48	0.23
Drinking	Age	0.00	0.06	0.81
	* Gender	0.02	4.50	0.04
	Presence of NSNSP	0.00	0.31	0.58
History of cardiovascular disease	Age	0.00	0.4	0.53
	* Gender	0.02	5.66	0.02 *
	Presence of NSNSP	0.00	0.27	0.61
Family history of cardiovascular disease	* Age	0.02	6.43	0.01 *
	Gender	0.00	0.06	0.8
	Presence of NSNSP	0.00	1.06	0.30

Legend: * statistically significant, *p* < 0.05.

**Table 3:** Pearson Correlation coefficients (r) and *p*-values for variables related to neck and shoulder pain severity (VAS: 34.90 ± 19.96).

Variable	Correlation (r)	*p*-Value
Age **	0.201	<0.01
BMI **	0.266	<0.01
Computer use (h/day)	−0.002	>0.05
Smartphone use (h/day)	−0.017	>0.05
Sitting position (h/day)	0.000	>0.05
Sleep (h/day)	−0.126	>0.05
Exercise (days/week)	0.090	>0.05
Exercise (h/day)	0.098	>0.05
Exercise (h/week)	0.081	>0.05
Aerobic (h/week)	0.133	>0.05

** A significant correlation *p* < 0.01.

## Data Availability

The data presented in this study are available upon reasonable request from the corresponding authors.

## Appendix A. Questions of the Survey in This Study

1. What is your age (in years)?
2. What is your gender?
3. What is your height (in cm)?
4. What is your weight (in kg)?
5. Do you have any recent (<3 months) injury to the neck or shoulder region?
6. Do you have any chronic (>3 months) injury to the neck or shoulder region?
7. Do you have any existing auto-immune diseases such as those listed below? (select all that applies)
8. Do you have other medical conditions/chronic illnesses? If yes, please state. -Selected Choice
9. What is your total duration of computer use in a day (hours)?
10. What is your total duration of smartphone use in a day (hours)?
11. On average, how many hours do you work or study in a sitting position daily?
12. On average, how many hours do you sleep daily?
13. How many cigarettes do you smoke daily? (in number of sticks) (Put 0 if you do not smoke)
14. Do you consume alcohol?
15. How many days do you exercise in a week?
16. How long do you exercise per day (hours)?
17. Presence of neck or shoulder pain (Yes/No)
18. Please rate your level of pain experienced in your neck and shoulder region. (0—no pain at all; 100—extreme and unimaginable pain ever experienced—Pain rating scale
19. Do you participate in regular sports activities or exercises?
20. How many hours do you participate in sports activities or exercise (include aerobic exercise) in a week?
21. Out of the total number of hours you exercise in a week, how many of those hours are aerobic exercises?
22. (Aerobic activities are activities that cause a noticeable increase in breathing rate and heart rate. This means that you will be able to carry on a conversation but will not have enough breath to sing. e.g.,: brisk walking/swimming/cycling/jogging/rowing/dancing etc.) If not applicable, put down 0
23. Do you have any history of cardiovascular diseases? For example, abnormal blood pressure, heart diseases, stroke etc.
24. Do your parents or siblings have any cardiovascular disease mentioned above?
25. Are you taking any medications this week? Put down the type of medication (e.g., Painkiller, anti-inflammatory).
